# Is Upregulation of Sarcolipin Beneficial or Detrimental to Muscle Function?

**DOI:** 10.3389/fphys.2021.633058

**Published:** 2021-03-01

**Authors:** Naresh C. Bal, Subash C. Gupta, Meghna Pant, Danesh H. Sopariwala, Geoffrey Gonzalez-Escobedo, Joanne Turner, John S. Gunn, Christopher R. Pierson, Scott Q. Harper, Jill A. Rafael-Fortney, Muthu Periasamy

**Affiliations:** ^1^School of Biotechnology, KIIT University, Bhubaneswar, India; ^2^Department of Physiology and Cell Biology, The Ohio State University, Columbus, OH, United States; ^3^Department of Biochemistry, Institute of Science, Banaras Hindu University, Varanasi, India; ^4^Departments of Microbiology and Microbial Infection and Immunity, The Ohio State University, Columbus, OH, United States; ^5^Texas Biomedical Research Institute, San Antonio, TX, United States; ^6^Center for Microbial Pathogenesis, Abigail Wexner Research Institute at Nationwide Children’s Hospital, Columbus, OH, United States; ^7^Department of Pathology and Laboratory Medicine, Nationwide Children’s Hospital, Columbus, OH, United States; ^8^Department of Pathology, The Ohio State University, Columbus, OH, United States; ^9^Department of Biomedical Education and Anatomy, The Ohio State University, Columbus, OH, United States; ^10^Department of Laboratory Medicine, Nationwide Children’s Hospital, Columbus, OH, United States; ^11^Burnett School of Biomedical Sciences, College of Medicine, University of Central Florida, Orlando, FL, United States

**Keywords:** sarcolipin, skeletal muscle, muscle disease, sarco/endo plasmic reticulum Ca^2+^ ATPase, Ca^2+^-handling proteins

## Abstract

Sarcolipin (SLN) is a regulator of sarco/endo plasmic reticulum Ca^2+^-ATPase (SERCA) pump and has been shown to be involved in muscle nonshivering thermogenesis (NST) and energy metabolism. Interestingly, SLN expression is significantly upregulated both during muscle development and in several disease states. However, the significance of altered SLN expression in muscle patho-physiology is not completely understood. We have previously shown that transgenic over-expression of SLN in skeletal muscle is not detrimental, and can promote oxidative metabolism and exercise capacity. In contrast, some studies have suggested that SLN upregulation in disease states is deleterious for muscle function and ablation of SLN can be beneficial. In this perspective article, we critically examine both published and some new data to determine the relevance of SLN expression to disease pathology. The new data presented in this paper show that SLN levels are induced in muscle during systemic bacterial (*Salmonella*) infection or lipopolysaccharides (LPS) treatment. We also present data showing that SLN expression is significantly upregulated in different types of muscular dystrophies including myotubular myopathy. These data taken together reveal that upregulation of SLN expression in muscle disease is progressive and increases with severity. Therefore, we suggest that increased SLN expression should not be viewed as the cause of the disease; rather, it is a compensatory response to meet the higher energy demand of the muscle. We interpret that higher SLN/SERCA ratio positively modulate cytosolic Ca^2+^ signaling pathways to promote mitochondrial biogenesis and oxidative metabolism to meet higher energy demand in muscle.

## Introduction

The sarco/endo plasmic reticulum Ca^2+^-ATPase (SERCA) plays a central role in skeletal muscle physiology by regulating cytosolic Ca^2+^-level. Studies have highlighted that futile SERCA pump activity modulated by sarcolipin (SLN) can be important in muscle nonshivering thermogenesis (NST; Morrissette et al., 2003; de Meis et al., 2005; Babu et al., 2007a,b; Kjelstrup et al., 2008; Bal et al., 2012, 2016). Recent structural analyses showed that SLN binds to SERCA pump in a transmembrane (TM) groove (Sahoo et al., 2013, 2015; Toyoshima et al., 2013; Winther et al., 2013) causing increased futile SERCA activity leading to heat production (Smith et al., 2002; Mall et al., 2006). By using the genetically altered mouse models, studies have demonstrated that SLN is a key mediator of muscle thermogenesis, especially NST (Bal et al., 2012, 2017; Maurya et al., 2015; Rowland et al., 2015a; Sopariwala et al., 2015). In small mammals (rodents), brown adipose tissue (BAT) is a major site of NST and is actively recruited both under cold as well as diet-induced thermogenesis (Enerback et al., 1997; Klingenspor, 2003; Cannon and Nedergaard, 2004; Nedergaard et al., 2005; Fromme and Klingenspor, 2011) employing uncoupling protein (UCP) 1 mediated heat production (Arechaga et al., 2001; Crichton et al., 2017; Klingenberg, 2017). The abundance of BAT is high in newborn babies (Bolton et al., 1970), but it becomes very limited or nonfunctional in adult humans (Cypess et al., 2009; Ouellet et al., 2012; Carpentier et al., 2018; Richard et al., 2020). Further, there are several species of mammals that lack BAT function due to mutation or loss of UCP1 gene during evolution like pigs, wild boars, horses, whales, elephants, and sea cows (Gaudry et al., 2017). Recent studies in wild boars by Nowack et al. (2017, 2019) demonstrated that SLN-mediated muscle NST plays a primary function during cold adaptation. Interestingly, studies in hibernating squirrels also showed that SLN expression correlates with sleep (down) and wake period (up; Oliver et al., 2019). Remarkably, birds do not have BAT, yet they maintain higher body temperature (≥40°C) and depend on skeletal muscle as the primary source of heat production *via* both shivering and NST (Bicudo et al., 2001; Teulier et al., 2014; Zheng et al., 2014a; Zhang et al., 2015). In several birds including ducklings and penguins, SR Ca^2+^-cycling has been implicated as the basis of muscle thermogenesis during cold adaptation (Duchamp et al., 1989, 1991, 1993; Dumonteil et al., 1993, 1994, 1995).

In addition to cold, SLN-mediated NST is also recruited during diet induced thermogenesis in mice (Bombardier et al., 2013a; Maurya et al., 2015). Loss of SLN in mice resulted in HFD induced obesity, whereas transgenic overexpression of SLN in the skeletal muscles increased basal metabolic rate and resistance against HFD-induced obesity (Maurya et al., 2015, 2018). Interestingly, SLN^OE^ mice did not show any muscle dysfunction, they exercised longer and showed higher fatigue resistance compared to SLN^−/−^ mice (Sopariwala et al., 2015). Exercise training has been shown to upregulate SLN expression in muscle suggesting a role for SLN in meeting increased energy demand (de Snoo, 2009). Despite recent progress, the functional relevance of altered SLN expression in muscle pathophysiology is not completely understood. Some studies have suggested that SLN upregulation in diseased muscle can be a contributing factor to muscle dysfunction especially in diseased muscle (Schneider et al., 2013; Voit et al., 2017; Campbell and Dicke, 2018; Niranjan et al., 2019). Therefore, the primary objective of this article is to explore the relevance of SLN expression in muscle pathophysiology, whether it is a cause or consequence of disease.

### Relevance of High SLN Expression in Neonatal Skeletal Muscle

Neonates are highly vulnerable to death due to hypothermia and effective thermogenesis increases the chances of their survival. As neonate muscles are not fully mature to sustain shivering in most mammals including rodents, their thermogenic demand is primarily reliant on NST mechanisms. SLN protein is detectable in muscle during gestational development but its level peaks around birth in all muscle types (Babu et al., 2007a; Pant et al., 2015a), which suggests that SLN mediated muscle NST plays a critical role in thermogenesis. SLN expression is high during the 1st week but is gradually decreased to low level by 21 days in the fast-twitch skeletal muscles. However, SLN continues to be expressed at high levels in fast oxidative and slow-twitch fibers in several muscles (diaphragm, soleus, atria, red gastrocnemius, masseter, trapezius, and tongue) throughout adult life (Pant et al., 2015a; Rowland et al., 2015b; Sopariwala et al., 2015). We found that gradual cold adaptation of neonatal mice prevents programmed developmental downregulation of SLN expression in adult quadriceps and gastrocnemius, glycolytic muscles (Pant et al., 2015a). Neonatal mice have significant amounts of BAT with very high UCP1 expression (Cannon and Nedergaard, 2004; Harris et al., 2020; Liu et al., 2020) and, therefore, it can be argued that the thermogenic demand of neonatal mice is met by BAT based NST. The finding that UCP1^−/−^ neonates are able to survive at 22°C (below thermoneutrality of 28°C) and can be gradually cold adapted to 4°C suggests the existence of additional NST components (Enerback et al., 1997). To further explore the role of SLN during neonatal NST, we generated double knockout (DKO) mice lacking UCP1 and SLN by breeding them either at 22°C or at 28°C. When bred at 22°C, (cold stress) the DKO offspring had higher mortality and were found to be below the expected Mendelian ratio. When bred at 28°C (thermoneutrality), the survival rate of DKO pups was close to the expected Mendelian ratio, indicating that SLN-mediated muscle NST is critical for survival of mice during their neonatal (Rowland et al., 2015a). Recent studies in pigs (which lack BAT-mediated NST) have shown that SLN and muscle NST are recruited during neonatal development of pigs (Nowack et al., 2017, 2019).

### SLN Based NST Is Hyper-Recruited When BAT Function Is Minimized

The existence of muscle NST has been reported in large adult mammals including dogs and humans (Davis, 1967; Hanssen et al., 2015; Blondin and Haman, 2018). However, the importance of muscle NST has been ignored since the mouse model was widely used as an experimental model for thermogenesis. Unlike large mammals, mice are endowed with significant amount of BAT masking the role of skeletal muscle NST (Bal et al., 2018). However, when BAT function is compromised (as found in UCP1^−/−^ and iBAT-ablated mice), the role of muscle based NST became obvious; remarkably, both UCP1^−/−^ and iBAT-ablated mice could be cold adapted to 4°C (Rowland et al., 2015a; Bal et al., 2016, 2017); even mild cold exposure of UCP1^−/−^ mice (16°C), a condition that does not evoke shivering increased SLN expression. Cold adaptation induced substantial remodeling in the skeletal muscles including increased mitochondrial abundance and expression of several markers of oxidative metabolism, this response was significantly blunted in the SLN^−/−^ littermates (Rowland et al., 2015a; Bal et al., 2017). Surprisingly, adaptation of SLN^−/−^ mice to mild cold (16°C) exhibited increased expression of UCP1 and other markers of BAT recruitment such as electron transport chain proteins and sympathetic innervation. Hence, loss of SLN in muscle is compensated by hyper-recruitment of BAT-based NST, suggesting that SLN-based thermogenesis is important even under mild cold (Bal et al., 2017). A recent study reported that cold adaptation in humans increased both insulin sensitivity and SLN expression (Hanssen et al., 2015). These studies suggest that both BAT and muscle-based NST are required for optimal thermogenesis and muscle NST can compensate for compromised BAT function as found in adult large mammals including pigs, wild boars, and humans.

### Induction of SLN Expression in Muscular Dystrophy and Atrophy

**Figure 1:**
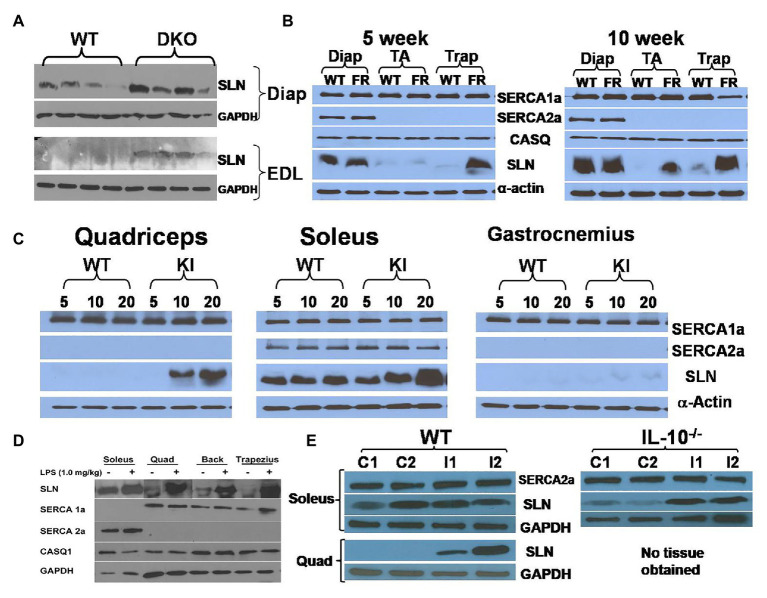
Sarcolipin (SLN) expression is upregulated in muscles in multiple disease states. **(A)** Representative western blots showing upregulation of SLN in extensor digitorum longus (EDL) and diaphragm muscles in Utrophin-dystrophin double knockout (DKO) mouse. Western blotting was performed using standard procedure as described before (Pant et al., 2015a). **(B)** Facioscapulohumeral muscular dystrophy (FSHD) region gene 1 (FRG1)-overexpression mice exhibit SLN upregulation in an age-dependent manner. FRG1-overexpression, wild-type, diaphragm, tibialis anterior, and trapezius are abbreviated as FR, WT, Diap, TA, and Trap, respectively. SLN upregulation increases with disease progression, whereas expression of SERCA1a, SERCA2a, and calsequestrin (CASQ) are not significantly affected. Methods for western blotting to detect the above proteins have been previously published by us (Gupta et al., 2009; Bal et al., 2012). **(C)** Muscles of myotubular myopathy 1 knock-in (MTM-KI) mice exhibit upregulation of SLN in an age-dependent manner. Age (in weeks) of mice studied is shown as numbers on the top of the western blots. MTM-KI is labeled as “KI.” Expression of SERCA1a and SERCA2a is unaltered. **(D)** Lipopolysaccharide (LPS) treatment cause fever in mice and lead to SLN upregulation in several muscles. Representative images from different western blots are pooled together. Expression of SERCA1a, SERCA2a, and CASQ1 are not altered. Muscle labeled as “Back” consists of several muscle groups from lower back portion of the mice. **(E)** *Salmonella* infection upregulate SLN expression in quadriceps muscle of WT mice and in soleus muscle of interleukin (IL) 10^−/−^ mice littermates. Control mice are labeled as C1 and C2. Mice infected with *Salmonella* are labeled as I1 and I2. TA, tibialis anterior; EDL, extensor digitorum longus; Trap, trapezius; Quad, quadriceps.

Sarcolipin upregulation is a consistent observation in several mouse models of muscle dysfunction with compromised muscle structure and contractile function (Schneider et al., 2013; Ravel-Chapuis et al., 2017; Voit et al., 2017; Fajardo et al., 2018; Liu et al., 2018; Tanihata et al., 2018; Niranjan et al., 2019; Wang et al., 2020). The majority of these studies explored SLN expression in the muscular dystrophy models generated by either gene deletion and/or mutations (Pant et al., 2015b; Rodrigues et al., 2016). In a utrophin-dystrophin DKO mouse model of Duchenne muscular dystrophy (DMD), we found upregulation of SLN expression in extensor digitorum longus (EDL), quadriceps, and diaphragm (Figure 1A and Supplementary Figure 1A). The degree of upregulation in the glycolytic muscles (EDL and quadriceps) was much greater than oxidative muscle like diaphragm, where SLN is abundant. Using the genetically altered mouse models of muscular dystrophy, Fajardo et al. (2018) reported that SLN deletion worsens dystrophic phenotype of the MDX mice, suggesting SLN upregulation as protective. Truncated dystrophin reintroduction by Tanihata et al. (2018) showed mitigation of dystrophic phenotype along with reduction in SLN expression, indicating SLN upregulation as a secondary event in DMD pathogenesis. Also, our studies in collaboration with Jeffrey Molkentin (University of Cincinnati) found that introduction of SLN-KO to MDX background does not improve the phenotype (unpublished data). On the other hand, Voit et al. (2017) reported that reduction of SLN expression improves DMD conditions and survival. Studies by Niranjan et al. (2019) suggested that SLN overexpression impairs myogenic differentiation of cultured muscle cells and silencing of SLN improves differentiation of dystrophic dog myoblasts. However, differentiating primary muscle cells and neonatal muscles are known to express significant levels of SLN protein (Babu et al., 2007a; Pant et al., 2015a; Maurya et al., 2018), indicating that it is not deleterious to muscle function.

Sarcolipin expression was also examined in a mouse model of facioscapulohumeral muscular dystrophy (FSHD; Gabellini et al., 2006). FSHD is caused by chromatin relaxation at human chromosome 4q35, leading to toxic upregulation of genes that would otherwise be epigenetically silenced in normal muscle. Although *DUX4* gene de-repression is now considered the primary insult underlying FSHD, several other candidate genes have been tested in cells and mice over the years, including *FRG1*. FRG1 is an evolutionarily conserved protein located in the 4q35 region that causes a severe myopathy when overexpressed at high levels in mouse muscle (Gabellini et al., 2006; Wallace et al., 2011). Interestingly, SLN expression was upregulated in trapezius muscle in a progressive manner: significantly higher upregulation was observed in 10-week old compared to 5-week old mice (Figure 1B). In TA muscle, upregulation was found in 10-week old FRG1 mice but not in 5-week old. However, SLN levels were not affected in diaphragm, where SLN expression is already abundant. Trapezius, that is most affected by the FSHD disease showed higher SLN levels, suggesting that it might not be the cause of the disease.

Another myopathy model studied is a mutant myotubularin knock-in mouse model (Pierson et al., 2012). Myotubularin is a phosphoinositide lipid phosphatase and loss of its activity leads to myotubular myopathy (MTM; Pierson et al., 2012; Lim et al., 2015). The knock-in (MTM1-KI) mouse carried a point mutation at R69C and exhibit mild progression of the myopathy with median life span of 66 weeks and we analyzed tissues taken at 5, 10, and 20 weeks of age. Quadriceps, soleus, and gastrocnemius exhibited progressive upregulation of SLN in the MTM1-KI mice (Figure 1C and Supplementary Figure 1B). Induction of SLN was prominent in quadriceps, a glycolytic muscle, while diaphragm (oxidative) did not show any appreciable upregulation. The finding that SLN upregulation was maximal in the most affected muscle (glycolytic) indicates that SLN upregulation might be compensatory to meet energy demand (metabolic adaptation of the muscle), since SLN is known to increase mitochondrial biogenesis and oxidative metabolism.

A common feature in all muscular dystrophies and other myopathies is altered mitochondrial metabolism and dysfunction (Bernardi and Bonaldo, 2013; Gan et al., 2018; Abrigo et al., 2019). In the utrophin-dystrophin DKO mice, the affected muscles showed distorted mitochondrial dynamics that impair metabolism (Pant et al., 2015b). In FSHD mouse models and patients, skeletal muscles show increased oxidative stress and abnormal mitochondrial function (Turki et al., 2012; Ansseau et al., 2017). In MTM, affected muscles exhibit abnormal mitochondrial positioning, shape, dynamics, and function (Pierson et al., 2012; Lim et al., 2015). In addition, in any myopathy, the structural architecture of muscles is impaired compromising the contractile efficiency leading to higher energy demand. The increase in SLN expression indicates a compensatory response to augment mitochondrial biogenesis and oxidative metabolism to meet the higher energy demand. Several recent studies in different types of muscle dystrophies showed upregulation of SLN expression (Ravel-Chapuis et al., 2017; Liu et al., 2018; Wang et al., 2020). Wang et al. (2020) created a new SLN^−/−^ mice model to test its role in a muscular dystrophy caused by Lamin A deficiency and found that genetic deletion of SLN enhances the disease process in Lmna^−/−^ which suggests that SLN upregulation is a protective mechanism, as a part of metabolic adaptation both in cardiac and skeletal muscles.

### SLN Expression in Muscle Atrophy

Studies from two different laboratories indicate a temporal upregulation of SLN expression with muscle atrophy. A hind limb immobilization study by Tomiya et al. (2019) showed that skeletal muscle atrophy upregulates SLN expression. Recent studies from Tupling’s lab suggest SLN upregulation in muscle opposes atrophy (Fajardo et al., 2017a,b). Fajardo et al. (2017a) showed that deletion of SLN in phospholamban (PLB, other better known SERCA regulator expressed predominantly in the heart) overexpression mouse increases atrophy of soleus muscle, suggesting a protective role for SLN. It is to be highlighted here that PLB overexpression causes a skeletal muscle phenotype similar to centronuclear myopathy; in contrast, SLN overexpression protects muscle from fatigue (Sopariwala et al., 2015). SLN is also upregulated in skeletal muscle of nebulin-KO mice that display accumulation of structurally abnormal mitochondria within myofibers (Ottenheijm et al., 2008).

### Role of SLN in Fever and Immune Response

Viral and bacterial infection is known to cause inflammatory response and hyperthermia: however, the mechanism of fever response is poorly understood. Recent studies suggest that BAT mediated thermogenesis is not recruited during fever (Eskilsson et al., 2020). We, therefore, investigated if SLN expression was altered in response to fever induced by lipopolysaccharide (LPS) treatment. LPS was administered intraperitoneally in a set of healthy wild type (WT) mice. Three days after treatment, SLN protein expression was analyzed in both glycolytic and oxidative muscles. Interestingly, SLN expression was induced in both fast glycolytic and oxidative skeletal muscles studied (Figure 1D), but there were no changes in the expression of SERCA 1a, 2a, and calsequestrin (CASQ) 1. To further explore the connection between SLN-based NST and fever, we examined whether *Salmonella* infection (a Gram-negative bacterium producing LPS) induces SLN in the skeletal muscles. We found that SLN expression was upregulated several folds in the quadriceps muscle, as compared to soleus, which express higher levels of SLN (enriched with oxidative fibers). Similarly, SLN expression was induced following *Salmonella* infection in interleukin (IL) 10 knockout mice in CBA/J genetic background (Figure 1E). IL-10 is known as a potent anti-inflammatory cytokine important in limiting pathogenic infection. These studies taken together suggest that SLN might play an important role in fever response; by increasing muscle thermogenesis and/or promote metabolic adaptation of muscle during fever, since prolonged fever can be energetically costly. The mechanism of SLN induction and its functional relevance during fever needs additional work and future studies should clarify this using the SLN^−/−^ and UCP1^−/−^ mouse models.

## Discussion and Future Perspectives

**Figure 2:**
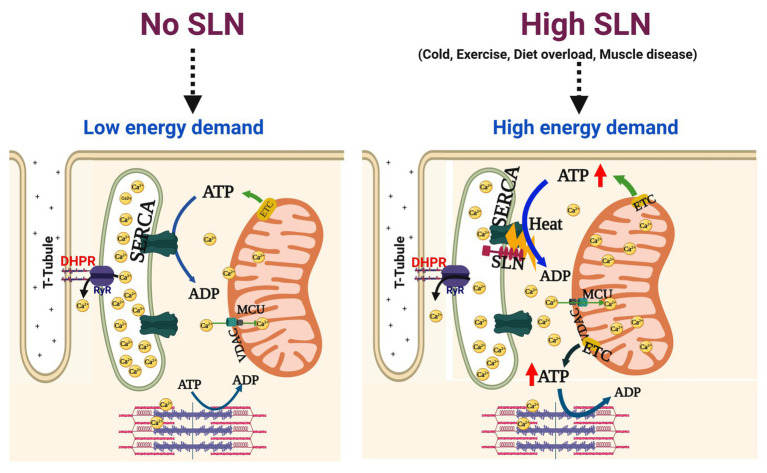
High SLN levels increase oxidative metabolism in the skeletal muscle. sarco/endo plasmic reticulum Ca^2+^-ATPase (SERCA) pump couples ATP hydrolysis to Ca^2+^ transport (1 ATP = 2 Ca^2+^) but this coupling is altered by SLN interaction with SERCA. When SLN is low or absent, SERCA efficiency is higher and no ATP is wasted, leading to lower energy demand. When SLN is abundant, it uncouples SERCA from Ca^2+^ transport causing futile cycling of SERCA and higher amount of ATP hydrolyzed, thus increasing the energy demand. At the same token, uncoupling of SERCA by SLN leads to elevation of cytosolic Ca^2+^ and ADP levels, both are strong activators of mitochondrial ATP synthesis, thereby helping in meeting the increased metabolic demand. Enhanced SLN activity plays an important role in muscle adaptation to high energy demand/expenditure such as cold/diet induced thermogenesis and endurance exercise that relies on mitochondrial oxidative metabolism. In addition, higher SLN expression/activity is beneficial to meet the increased energy demand in structurally compromised dystrophic muscles. MCU, mitochondrial uniporter; ETC, electron transport chain; VDAC, voltage dependent anion channel.

Sarcolipin is an important regulator of SERCA pump in both cardiac and skeletal muscle. However, its role in muscle patho-physiology is still an evolving topic. It has also become a topic of interest, whether SLN upregulation during muscle dystrophy/atrophy disease is detrimental or beneficial to muscle function. In this perspective article, we critically examined several studies carried out in the muscle disease models where SLN expression is upregulated and as well data from an SLN over-expression mouse model. The studies from the SLN over-expression mouse model suggest that higher levels of SLN expression in adult life do not cause muscle pathology but can be beneficial to muscle function including exercise capacity. The SLN^OE^ mice remained healthy and maintained better muscle health even after 2 years (Bal et al., unpublished data). Studies using the genetically altered mouse models have shown that SLN plays a central role in promoting fat utilization and oxidative metabolism in muscle (Maurya and Periasamy, 2015; Pant et al., 2016; Periasamy et al., 2017; Bal et al., 2018). These studies suggest that SLN acts as a dual regulator of energy metabolism as schematically shown in Figure 2: (a) by uncoupling SERCA, it increases ATP utilization creating energy demand and (b) by activating Ca^2+^-signaling, it promotes mitochondrial biogenesis and ATP production (Smith et al., 2002; Babu et al., 2007a,b; Bal et al., 2012; Bombardier et al., 2013a,b; Sahoo et al., 2013, 2015; Maurya et al., 2015, 2018; Paran et al., 2015; Gamu et al., 2020). In addition studies conducted in several dystrophic mouse models suggest that the induction of SLN expression is progressive (Figure 1) and is a consequence of the disease. Further hypothyroidism, a condition that compromise recruitment of BAT function, significantly induces SLN expression in the skeletal muscle and suggested that SLN is important for adaptive thermogenesis (Kaspari et al., 2020). Therefore, upregulation of SLN should not be viewed as the cause of the disease; rather, it is a compensatory response to meet the increased thermogenic and metabolic demand in muscle.

Although numerous studies have shown that SLN gene/protein expression is altered in muscle pathophysiology, the mechanisms that regulate SLN gene/protein expression have not been addressed. Conditions that increase SLN expression are closely linked with increased mitochondrial biogenesis/dynamics, oxidative metabolism, and fat utilization. It is also conceivable that similar mechanisms that regulate SLN expression during cold stress are also recruited in pathological states. It is presently unclear whether disease states activate inflammatory cytokines that might along with other energy sensors, increase SLN expression. Future research undoubtedly needs to explore what factors induce SLN expression and how its levels can be upregulated. There is also significant difference in the pattern of SLN expression in mice vs. large mammals. Unfortunately, majority of the studies thus far have primarily relied on the mouse models that contain significantly higher amount of glycolytic fibers than oxidative. Unlike rodents, SLN expression is at least 10-fold higher in large mammals (having larger proportion of oxidative fibers) including humans which suggest that it is essential for muscle physiology. Future research should be focused on large mammals to better define the relevance of SLN in muscle pathophysiology.

Two other poorly studied physiological conditions where SLN might be involved are fear-induced hyperthermia and postprandial heat production. Fear induced thermogenesis has been shown to be BAT-independent and depend on skeletal muscle, employing β-adrenoceptors (Marks et al., 2009). A recent study has shown that exposure of predator odor (trigger fear without other stress) induce skeletal muscle NST *via* β-adrenergic receptors of the sympathetic nervous system, which provides resistance to fatigue, altering fuel selection (Gorrell et al., 2020); these conditions that are known to activate SLN-based functions. Interestingly, some preliminary studies have indicated that SLN mRNA is upregulated in brain-induced muscle thermogenesis, suggesting that SLN mediated NST might be neurally recruited (Titus et al., 2017; Gibson et al., 2019). However, the detailed role of SLN in fear-induced muscle thermogenesis is yet to be illustrated. Postprandial thermogenesis also recruits skeletal muscle associated with augmented mitochondrial metabolism and increased expression of RyR1 and SERCA2a (Henry et al., 2008; Clarke et al., 2012). These conditions are linked to SLN-based thermogenesis during cold adaptation (Bal et al., 2016). SLN is also expressed in the myocardium but the functional relevance is not completely understood. In the healthy heart, SLN is expressed abundantly in the atria but it is very low in the ventricle (Minamisawa et al., 2003; Vangheluwe et al., 2005; Babu et al., 2007a). Intriguingly, during several different cardiac pathologies, including heart failure, SLN expression is induced in both atria and ventricle (Zheng et al., 2014b; Morales Rodriguez et al., 2020). These data suggest that SLN expression is increased in the failing heart, which is energy starved and SLN upregulation might play a compensatory role to increase oxidative mitochondrial metabolism. Answer to these questions will provide insight to define role of SLN in the disease conditions. These findings will help in targeting muscle metabolism to counter muscle pathologies as well as metabolic syndromes like obesity and type 2 diabetes.

## Author Contributions

NB and MPe conceived idea, prepared the figures, and wrote the first draft. SG, DS, and MPa performed the experiments and analyzed the data. GG-E, JT, JG, CP, SH, and JR-F designed the mice study and performed the animal experiments. All authors contributed to the article and approved the submitted version.

### Conflict of Interest

The authors declare that the research was conducted in the absence of any commercial or financial relationships that could be construed as a potential conflict of interest.
