# Supplementary material for: Is Upregulation of Sarcolipin Beneficial or Detrimental to Muscle Function?
Source: Front Physiol. 2021 Mar 1;12:633058. doi: 10.3389/fphys.2021.633058 (PMC7956958; doi:10.3389/fphys.2021.633058)
Supplement: Supplementary file 1 [file Table_1.docx]

**
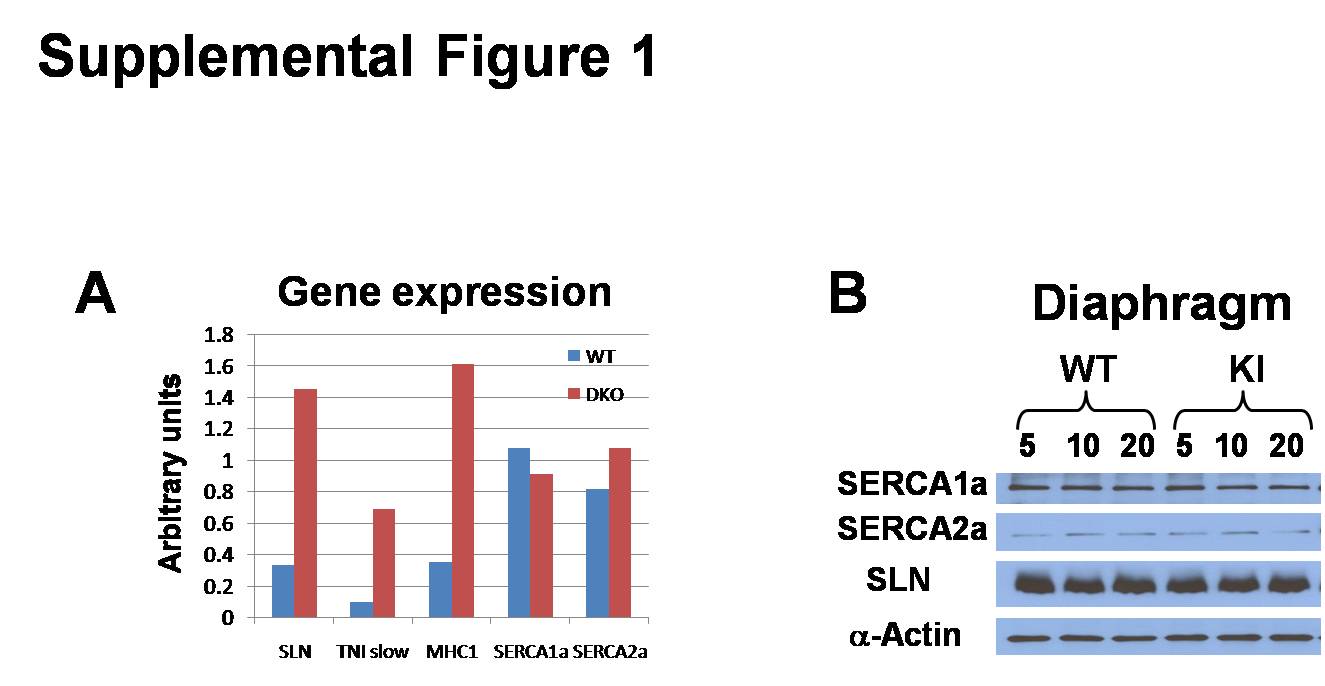
**

**SUPPLEMENTAL FIGURE 1: Upregulation of SLN expression in muscles during diseases. (A)** Gene expression analyzed using routine RT-PCR in quadriceps muscle in Utrophin-dystrophin double knockout (DKO) mouse. TNI slow, MHC1 stand for Slow twitch skeletal muscle isoform of troponin I and Myosin heavy chain 1 respectively. **(B)** Representative western blots of whole tissue homogenate from diaphragm muscles of Myotubular myopathy 1 knock-in (MTM-KI) mice.
